# Blood Cell Count Derived Inflammation Indexes in Patients with Idiopathic Pulmonary Fibrosis

**DOI:** 10.1007/s00408-020-00386-7

**Published:** 2020-08-25

**Authors:** Angelo Zinellu, Panagiotis Paliogiannis, Elisabetta Sotgiu, Sabrina Mellino, Arduino A. Mangoni, Elisabetta Zinellu, Silvia Negri, Claudia Collu, Gianfranco Pintus, Antonello Serra, Angelo Maria Pistuddi, Ciriaco Carru, Pietro Pirina, Alessandro G. Fois

**Affiliations:** 1 Department of Biomedical Sciences, University of Sassari, Sassari, Italy; 2 Department of Medical, Surgical and Experimental Sciences, University of Sassari, Sassari, Italy; 3 Unit of Respiratory Diseases, University Hospital Sassari (AOU), Sassari, Italy; 4 Department of Clinical Pharmacology, College of Medicine and Public Health, Flinders University and Flinders Medical Centre, Adelaide, Australia; 5 Department of Medical Laboratory Sciences, College of Health Sciences and Sharjah Institute for Medical Research, University of Sharjah, P.O. Box: 27272, Sharjah, United Arab Emirates; 6 Unit of Occupational Medicine, University Hospital Sassari (AOU), Sassari, Italy

**Keywords:** Idiopathic pulmonary fibrosis, IPF, Inflammation, LMR, NLR, PLR

## Abstract

**Purpose:**

Inflammation and immunity play a pivotal but yet unclear role in idiopathic pulmonary fibrosis (IPF), a chronic disorder characterized by progressive damage of lung parenchyma and severe loss of lung function despite optimal treatment. However, the pathophysiological and predictive role of combined blood cell count indexes of inflammation in IPF is uncertain.

**Methods:**

Seventy-three patients with IPF and 62 healthy subjects matched for age, gender and smoking status were included in this cross-sectional study.

**Results:**

We found significant differences in neutrophil to lymphocyte ratio (NLR), derived neutrophil to lymphocyte ratio (dNLR), monocyte to lymphocyte ratio (MLR), platelet to lymphocyte ratio (PLR), systemic inflammation response index (SIRI) and aggregate index of systemic inflammation (AISI) between IPF patients and healthy controls. In logistic regression, all combined blood inflammation indexes, barring PLR, were independently associated with the presence of IPF after adjusting for age, gender, body mass index and smoking status. Furthermore, significant associations between FVC% and NLR, LMR, SIRI and AISI, and between DLCO% and NLR, dNLR, LMR, SIRI and AISI, were observed.

**Conclusions:**

In conclusion, our data indicate significant alterations of combined blood cell count indexes of inflammation in IPF.

## Introduction

Idiopathic pulmonary fibrosis (IPF) is a chronic progressive fibrosing interstitial lung disease of unknown aetiology, occurring mainly in adults, that is characterized by a histopathologic and/or radiologic pattern of usual interstitial pneumonia [1]. The progressive accumulation of fibrotic tissue leads to irreversible lung damage. As a consequence, the estimated survival of untreated patients is limited, 2–3 years [2]. It is commonly accepted that fibrogenesis in IPF results from an aberrant repair process due to recurrent microinjury of alveolar epithelial cells [3–5]. The exaggerated wound repair and tissue remodelling lead to chronic inflammation and, ultimately, fibrosis. Both innate and adaptative responses seem to be involved in this process [6]. The excessive inflammatory response in IPF is multifactorial although the transforming growth factor-β (TGF-β) signalling is thought to play a key role [7]. It is well known that inflammation involves a highly coordinated network of immune-related cells, such as neutrophils, lymphocytes and monocytes [8]. Platelets can also modulate inflammation through direct cell–cell communications and the production of inflammatory mediators [9].

There is good evidence that indexes derived from calculating the ratios between neutrophils, lymphocytes, monocytes and platelets counts are more strongly associated with chronic inflammation conditions when compared with individual cell populations [8]. Indeed, combined blood cell count indexes of systemic inflammation have gained relevant scientific interest over the last decade. The neutrophil to lymphocyte ratio (NLR), derived neutrophil to lymphocyte ratio (dNLR), monocyte to lymphocyte ratio (MLR), platelet to lymphocyte ratio (PLR), systemic inflammation response index (SIRI) and aggregate inflammation systemic index (AISI) are increasingly being investigated as markers of inflammation in several disorders [10–14], including lung disease states such as COPD [15, 16] and asthma [17]. However, no information is currently available regarding possible associations between combined blood cell count indexes of inflammation and IPF. We sought to address this issue by measuring NLR, dNLR, PLR, LMR SIRI and AISI in IPF patients and in controls and investigating associations with clinically relevant parameters of lung function, FEV_1_ (Forced Expiratory Volume in the 1st second), FVC (Forced Vital Capacity) and DLCO (Diffusion Capacity for Carbon Monoxide).

## Materials and Methods

This was a case–control study of 73 consecutive newly diagnosed IPF patients recruited at the Respiratory Unit of the University of Sassari between 2016 and 2019. All patients signed a written consent and the study was approved by the ethics committee of the University Hospital of Cagliari (2262/CE-17/11/2015). IPF was diagnosed according to the current evidence-based guidelines for the diagnosis and management of IPF [18]. High-resolution computed tomography (HRCT) images and lung-biopsy samples of individual patients were reviewed by two experienced radiologists and two experienced pathologists, respectively. Each diagnosis was confirmed during multidisciplinary meetings attended by local respiratory, pathology, and radiology experts in interstitial lung disease. Measurements of FEV_1_, FVC and DLCO, expressed as percentages of predicted values (%FEV_1_, %FVC and %DLCO, respectively), were performed in accordance with the criteria published by the American Thoracic Society and the European Respiratory Society [19]. Patients with the following conditions were excluded from the study: patients currently experiencing an acute exacerbation of IPF; comorbid conditions including malignancy, bleeding tendency, and severe hepatic or renal dysfunction; use of immunosuppressants, antifibrotic drugs including interferon, d-penicillamine, and colchicine, or oral corticosteroids during the preceding 3 months. A group of 63 healthy controls matched for age, sex and smoking status, with no medical history, was also included.

Fasting blood samples from IPF patients and matched controls were respectively collected at first hospital admission in the Unit of Respiratory Diseases, and the Unit of Occupational Medicine of the University Hospital of Sassari (AOU Sassari); blood cell counts were performed using an automatic blood counter CellDyn Sapphire (Abbott Diagnostics, Santa Clara, CA, USA) in a certified laboratory. The following combined indexes were evaluated: NLR, dNLR, LMR, PLR, SIRI and AISI. The SIRI was calculated by multiplying the number of neutrophils with that of monocytes and dividing the product by the number of lymphocytes [20]. The AISI was calculated by multiplying the number of neutrophils, monocytes and platelets and dividing the product by the number of lymphocytes [10].

Results are expressed as mean values (mean ± SD) or median values (median and interquartile range, IQR). Variables distribution was assessed by the Shapiro–Wilk test. Statistical differences between groups were evaluated using unpaired Student’s t-test or Mann–Whitney rank sum test, as appropriate. Differences between categorical variables were assessed by Chi-squared test. Correlations between variables were assessed by Pearson’s correlation or Spearman’s correlation as appropriate. Logistic regression analysis was performed to evaluate independent associations between IPF disease and combined blood cell count indexes of inflammation. The association between different indexes and IPF was evaluated using receiver operating characteristics (ROC) curve analysis and selection of optimal cut-off values for sensitivity and specificity according to the Youden Index. Statistical analyses were performed using MedCalc for Windows, version 15.4 64 bit (MedCalc Software, Ostend, Belgium).

## Results

Table 1 describes the clinical characteristics, lung functional parameters, and blood cell counts of IPF patients and healthy controls. As expected, FEV_1_ (%) and FVC (%) were significantly lower in IPF patients than in controls (mean 81 ± 22% vs 92 ± 18%, *p* < 0.001; median 72%; IQR 58–90% vs 92%; IQR 84–105% *p* < 0.001, respectively). IPF patients had higher WBCs (median 8.7 × 10^9 ^L; IQR 7.3–10.3 × 10^9 ^L vs 7.2 × 10^9 ^L; IQR 6.4–8.7 × 10^9 ^L, *p* < 0.001) and neutrophils (median 5.5 × 10^9 ^L; IQR 4.6–6.7 × 10^9 ^L vs 4.1 × 10^9 ^L; IQR 3.5–5.4 × 10^9 ^L, *p* < 0.001) and lower lymphocyte values (median 2.0 × 10^9 ^L; IQ 1.5–2.4 × 10^9 ^L vs 2.3 × 10^9 ^L; IQR 1.9–2.6 × 10^9 ^L, *p* < 0.05). There were no significant between-group differences in BMI, monocyte and platelet values.

**Table 1:** Demographic and functional parameters and blood count results of healthy subjects and IPF patients

	Controls (*n* = 62)	IPF (*n* = 73)
Age (years)	67 (66–69)	70 (64–75)
Gender (F/M)	19/43	22/51
BMI (kg/m^2^)	27.1 ± 4.0	27.2 ± 3.9
Smokers, never/current/ex (%)	30/10/60	19/6/75
FEV_1_ (% predicted)	99 ± 18	81 ± 22^***^
FVC (% predicted)	92 (84–105)	72 (58–90)^***^
TLC (% predicted)	–	75 ± 17
DLCO (% predicted)	–	55 ± 23
6MWT (m)	–	342 ± 206
GAP index (1/2/3/4/5/6/7%)	–	4/19/25/24/17 /8/3
Disease stage (I/II/III %)	–	48/42/10
WBC (× 10^9^ L)	7.2 (6.4–8.7)	8.7 (7.3–10.3)^***^
Monocytes (× 10^9^ L)	0.5 (0.39–0.60)	0.5 (0.40–0.70)
Lymphocytes (× 10^9^ L)	2.3 (1.9–2.6)	2.0 (1.5–2.4)^*^
Neutrophils, (× 10^9^ L)	4.1 (3.5–5.4)	5.5 (4.6–6.7)^***^
Platelet (× 10^9^ L)	242 ± 60	242 ± 68

Data are presented as mean ± standard deviation or median (interquartile range)

*FEV1* forced expiratory volume in the 1st second, *FVC* forced vital capacity, *TLC* total lung capacity, *DLCO* diffusion capacity for carbon monoxide, *GAP* gender, age and two lung physiology variables index, *P6MWT* six-minute walk test, *WBC* white blood cell

*p < 0.05, **p < 0.01, ***p < 0.001

The combined indexes data are described in Table 2. NLR (median 2.67; IQR 1.89–2.67 vs 1.87; IQR 1.52–2.35, *p* < 0.001), d-NLR (median 1.88; IQR 1.33–2.26 vs 1.31; IQR 1.15–1.70, *p* < 0.001), PLR (mean 132 ± 74 vs 110 ± 36, *p* < 0.05), SIRI (median 1.49; IQR 0.89–2.40 vs 0.89; IQR 0.57–1.26, *p* < 0.001) and AISI (median 335; IQR 183–550 vs 198; IQR 128–316, *p* < 0.001) were significantly higher in IPF patients whereas the LMR was significantly lower (mean: 4.05± 1.75 vs 5.10 ± 1.71, *p* < 0.001). In univariate logistic regression, crude ORs for all inflammation combined indexes were significant, as reported in Table 3. In multivariate logistic regression, the ORs for NLR (OR = 2.633; 95% CI 1.604–4.322, *p* = 0.0001), d-NLR (OR = 3.941; 95% CI 1.851–8.390, *p* = 0.0004), LMR (OR = 0.685; 95%CI 0.543–0.865, *p* = 0.0015), SIRI (OR = 3.840; 95%CI 1.906–7.736, *p* = 0.0002) and AISI (OR = 1.003; 95% CI 1.001–1.005, *p* = 0.0045) remained significant after adjusting for age, gender, BMI and smoking status.

**Table 2:** Complete blood cell count-derived indexes in idiopathic pulmonary fibrosis and healthy subjects

	Controls (62)	IPF (73)
NLR	1.87 (1.52–2.35)	2.67 (1.89–2.67)^***^
dNLR	1.31 (1.15–1.70)	1.88 (1.33–2.26)^***^
LMR	5.10 ± 1.71	4.05 ± 1.75c
PLR	110 ± 36	132 ± 74^*^
SIRI	0.89 (0.57–1.26)	1.49 (0.89–2.40)^***^
AISI	198 (128–316)	335 (183–550)^***^

Data are presented as mean ± standard deviation or median (interquartile range)

*NLR* neutrophil to lymphocyte, *dNLR* derived NLR, *LMR* lymphocyte to monocyte, *PLR* platelet to lymphocyte, *SIRI* Systemic Inflammation Response Index, *AISI* aggregate index of systemic inflammation, *IPF* idiopathic pulmonary fibrosis

*p < 0.05, **p < 0.01, ***p < 0.001

**Table 3:** Univariate and multivariate logistic regression for blood cell count–derived indexes

		95% CI	*p*
Crude OR			
NLR	2.851	1.748–4.649	< 0.0001
dNLR	4.486	2.126–9.466	0.0001
LMR	0.697	0.559–869	0.0013
PLR	1.008	1.000–1.015	0.043
SIRI	3.793	1.972–7.297	0.0001
AISI	1.003	1.001–1.005	0.0014
Corrected OR			
NLR	2.633	1.604–4.322	0.0001
dNLR	3.941	1.851–8.390	0.0004
LMR	0.685	0.543–0.865	0.0015
PLR	1.007	0.999–1.015	0.10
SIRI	3.840	1.906–7.736	0.0002
AISI	1.003	1.001–1.005	0.0045

*NLR* neutrophil to lymphocyte, *dNLR* derived NLR, *LMR* lymphocyte to monocyte, *PLR* platelet to lymphocyte, *SIRI* Systemic Inflammation Response Index, *AISI* aggregate index of systemic inflammation, *OR* odds ratio, *CI* confidence interval

In IPF patients, combined blood cell count indexes of inflammation were also significantly associated with functional lung parameters (Table 4). In particular, FEV_1_ was negatively associated with NLR (rho = − 0.24, *p* = 0.04), SIRI (rho = − 0.27, *p* = 0.019) and AISI (rho = − 0.26, *p* = 0.03), FVC% was negatively associated with NLR (rho = − 0.32, *p* = 0.007), SIRI (rho = − 0.30, *p* = 0.012) and AISI (rho = − 0.24 *p* = 0.04) and positively related with LMR (rho = 0.27, *p* = 0.02), and DLCO% was negatively associated with NLR (rho = − 0.40, *p* = 0.003), dNLR (rho = − 0.33, *p* = 0.015), SIRI (rho = − 0.35, *p* = 0.01) and AISI (rho = − 0.16, *p* = 0.26), and positively associated with LMR (rho = 0.28, *p* = 0.04). By contrast, barring LMR and SIRI, there were no significant associations between combined blood cell count indexes and functional lung parameters in healthy controls.

**Table 4:** Correlations between blood cell count indexes of systemic inflammation and functional parameters in patients with IPF and controls

	Controls (*n* = 62)		IPF (*n* = 73)		
	FVC (%)	FEV_1_ (%)	FVC (%)	FEV_1_ (%)	DLCO (%)
NLR	rho = 0.04 *p* = 0.74	rho = −0.07 *p* = 0.60	rho = −0.32 *p* = 0.007	rho = −0.24 *p* = 0.04	rho = −0.40 *p* = 0.003
dNLR	rho = 0.08 *p* = 0.52	rho = 0.00 *p* = 0.96	rho = −0.18 *p* = 0.11	rho = −0.12 *p* = 0.31	rho = −0.33 *p* = 0.015
LMR	rho = 0.28 *p* = 0.03	rho = 0.39 *p* = 0.002	rho = 0.27 *p* = 0.02	rho = 0.22 *p* = 0.06	rho = 0.28 *p* = 0.04
PLR	rho = 0.21 *p* = 0.12	rho = 0.15 *p* = 0.24	rho = −0.19 *p* = 0.11	rho = −0.13 *p* = 0.27	rho = −0.16 *p* = 0.26
SIRI	rho = −0.24 *p* = 0.06	rho = −0.30 *p* = 0.017	rho = −0.30 *p* = 0.012	rho = −0.27 *p* = 0.019	rho = −0.35 *p* = 0.01
AISI	rho = −0.16 *p* = 0.21	rho = −0.25 *p* = 0.05	rho = −0.24 *p* = 0.04	rho = −0.26 *p* = 0.03	rho = −0.29 *p* = 0.04

*NLR* neutrophil to lymphocyte, *dNLR* derived NLR, *LMR* lymphocyte to monocyte, *PLR* platelet to lymphocyte, *SIRI* Systemic Inflammation Response Index, *AISI* Aggregate index of systemic inflammation, *IPF* idiopathic pulmonary fibrosis, *FVC* forced vital capacity, *FEV1* forced expiratory volume in the 1st second, *DLCO* diffusion capacity for carbon monoxide

ROC curve analysis was carried out to evaluate the sensitivity, specificity, and the diagnostic accuracy of the combined inflammation indexes in distinguishing IPF patients from healthy subjects (Table 5). Except for PLR, all AUC values were significant. The better performing index was the NLR, with a threshold of 2.545, 55% sensitivity, and 85% specificity (AUC = 0.735, 95% CI 0.652–0.807 *p* < 0.001).

**Table 5:** Receiver operating characteristics (ROC) curves and prognostic accuracy of blood cell count–derived indexes

	AUC	95% CI	*p*-value	Cut-off	Sensibility (%)	Specificity (%)
NLR	0.735	0.652–0.807	< 0.0001	> 2.545	55	85
dNLR	0.714	0.630–0.788	< 0.0001	> 1.862	53	85
LMR	0.663	0.576–0.742	0.0004	< 2.857	29	100
PLR	0.590	0.502–0.674	0.07	> 113	56	66
SIRI	0.729	0.646–0.802	< 0.0001	> 1.333	58	85
AISI	0.685	0.599–0.762	0.0001	> 272	60	71

*NLR* neutrophil to lymphocyte, *dNLR* derived NLR, *LMR* lymphocyte to monocyte, *PLR* platelet to lymphocyte, *SIRI* Systemic Inflammation Response Index, *AISI* Aggregate index of systemic inflammation, *AUC* area under the curve, *CI* confidence interval

## Discussion

We observed significant differences in neutrophil to lymphocyte ratio (NLR), derived neutrophil to lymphocyte ratio (dNLR), monocyte to lymphocyte ratio (MLR), platelet to lymphocyte ratio (PLR), systemic inflammation response index (SIRI) and aggregate index of systemic inflammation (AISI) between patients with IPF and a group of healthy controls matched for age, gender and smoking status. All combined blood inflammation indexes, except for PLR, were independently associated with IPF after adjusting for age, gender, body mass index and smoking status. Furthermore, there were significant associations between FVC% and NLR, LMR, SIRI and AISI, and between DLCO% and NLR, dNLR, LMR, SIRI and AISI.

IPF is the most severe and frequent form of interstitial lung disease, characterized by aberrant deposition of extracellular matrix due to recurrent micro-injuries to the alveolar epithelium. Mediators released by epithelial cells activate fibroblast proliferation leading to accumulation of fibroblasts and highly active myofibroblasts. These cells are resistant to apoptosis and lead to extensive lung remodelling, through deposition of extracellular matrix components such as hyaluronan, fibronectin and interstitial collagens, and consequent alveolar wall thickening and impaired gas exchange [6, 21]. While the exact aetiology of IPF remains unknown, several lines of evidence indicate that all stages of fibrosis are accompanied by innate and adaptive response, although treatment that modulates inflammation, e.g. steroids, has been shown to be ineffective, or even harmful, in clinical trials [22–24]. Although this has led to the proposition that inflammation may represent an epiphenomenon, rather than a key driver in IPF, the active involvement of immune cells in the pathophysiology of the disease is well accepted.

Recent findings suggest that neutrophil elastase (NE) promotes TGF-β activation, fibroblast proliferation and myofibroblast differentiation, all contributing to enhance fibrosis [25]. The same study, according to previous observations [26], demonstrated that NE antagonists attenuate fibrosis both in asbestos and bleomycin induced fibrosis models, thus confirming the pro-fibrotic role of NE in IPF. Monocytes also play a crucial role during fibrogenesis as progenitor cells for pro-fibrotic macrophages and fibrocytes and by releasing pro-fibrotic inflammatory cytokines [7]. Furthermore, macrophages exhibit tissue-regenerating and pro-fibrotic function, depending on the local cytokine environment. In particular, M2 macrophages seem to have a central role in fibrosis regulation [27] acute exacerbations of IPF [28]. In addition, adaptive immunity cells Th1 and Th17 promote pulmonary fibrosis, whereas Th2 and Th22 cells inhibit fibrosis. Similarly, regulatory T cells (Tregs) and Th 9 cells exhibit both pro- and anti-fibrotic characteristics [29]. These observations strongly justify the investigation of blood cell count derived inflammation indexes in IPF.

Our results show significant alterations of these indexes in IPF patients, thus supporting the ability of these markers to detect systemic inflammation also in this group. These data agree with previous studies demonstrating that NLR and PLR are reliable indexes of systemic inflammation and predict the prognosis of several chronic inflammatory diseases [10, 11, 15–17]. In logistic regression, except for PLR, all combined blood inflammation indexes were independently associated with the presence of IPF after adjusting for age, gender, BMI and smoking status. Moreover, they were significantly associated with robust functional lung parameters. In particular, the association between FVC% and NLR, LMR, SIRI and AISI and between DLCO% and NLR, dNLR, LMR, SIRI and AISI indicate a strong relationship between these inflammation indexes and disease severity. These data agree with previous observations in which the concentration of inflammatory biomarkers, such as BALF IL-8, was significantly associated with a reduced FVC% and DLCO% [30], and the extent of sputum neutrophilia was correlated with FVC% in IPF patients [31]. Furthermore, Tsoutsou et al. [32] found that serum concentrations of ICAM-2 (a circulating adhesion molecule with immunomodulatory effects) were negatively associated with DLCO%. Daniil et al. [33] also that reported that tissue CD8 + TLs inversely correlated with FVC%, TLC% and DLCO% and that CD68 + cells negative correlated with both FVC% and FEV1%.

In ROC curve analysis, the NLR, dNLR, LMR, SIRI and AISI were able to significantly discriminate patients with IPF from healthy subjects. The diagnostic accuracy was higher (AUC > 0.7) with NLR, dNLR and SIRI. NLR was the index which performed better, even than the combined indexes (SIRI and AISI) which included more than two blood cell populations in their calculation. The rationale for the use of such indexes, is based on the fact that multiple blood cell populations participate in systemic inflammation as we mentioned before, and therefore, inclusion of these populations in the calculation of an index should better reflect the inflammatory state and improve its predictive abilities. Indeed, the activation of neutrophils during systemic inflammation, not only increases their number, but stimulates also the production of several mediators (like IL-1a), which in turn stimulate megakaryocytes to produce platelets [34]; in addition, specific negligible fractions of monocytes in steady-state conditions have been reported to consistently increase during inflammation [35, 36]. Therefore, AISI or SIRI are hypothesized to perform better than more restricted indexes. Nevertheless, our study showed that both platelet and monocytes are not different between IPF patients and healthy controls, and combined indexes do not perform better than NLR. The latters have shown better predictive abilities than NLR, in acute clinical conditions and malignancies [10, 14, 37, 38]; this probably means that in cases of chronic inflammation adaptive phenomena prevent the increment of specific other than neutrophil and lymphocyte cell populations and stabilize their turnover. As a result, combined indexes may not be more effective than NLR in chronic systemic inflammation. The issue needs further evaluation. In addition, given the relatively low specificity of all the indexes tested in this study towards individual disease states, additional studies are required to determine their role in diagnosis, risk stratification, and prognosis, and their capacity to discriminate between IPF and other forms of interstitial lung diseases [15–17].

Our study has some limitations. In particular, the relatively small sample size and the single centre nature of the study, even accounting for the low prevalence of the disease, limits its generalizability. Furthermore, the cross-sectional design did not allow establishing a definite cause-effect relationship between the studied indexes, the presence of IPF, and the lung function parameters and no correlations were analysed between these indexes and relevant clinical parameters, such as dyspnoea and the six-minute walking test. These issues notwithstanding, our data show the presence of significant alterations in NLR, dNLR, LMR, SIRI and AISI values in IPF patients when compared to healthy controls, and clinically relevant associations with lung function parameters. Such indexes might be attractive from a clinical point of view as their calculation is relatively simple, inexpensive, and part of routinely available laboratory investigations. Nevertheless, large, prospective studies are now required to investigate their prognostic role in IPF.

## Data Availability

Data can be provided by the corresponding author upon reasonable request.
